# Effect of nocturnal EPAP titration to abolish tidal expiratory flow limitation in COPD patients with chronic hypercapnia: a randomized, cross-over pilot study

**DOI:** 10.1186/s12931-020-01567-x

**Published:** 2020-11-18

**Authors:** Emanuela Zannin, Ilaria Milesi, Roberto Porta, Simona Cacciatore, Luca Barbano, R. Trentin, Francesco Fanfulla, Michele Vitacca, Raffaele L. Dellacà

**Affiliations:** 1 Dipartimento di Elettronica, Informazione e Bioingegneria, Politecnico di Milano, Milano, Italy; 2 Istituti Clinici Scientifici Maugeri IRCCS, Respiratory Rehabilitation of the Institute of Lumezzane, Brescia, Italy; 3 Istituti Clinici Scientifici Maugeri IRCCS, Respiratory Function and Sleep Medicine Unit of the Institute of Pavia, Pavia, Italy

**Keywords:** Non-invasive ventilation, Chronic obstructive pulmonary disease, Forced oscillation technique, Intrinsic PEEP

## Abstract

**Background:**

Tidal expiratory flow limitation (EFL_T_) promotes intrinsic PEEP (PEEPi) in patients with chronic obstructive pulmonary disease (COPD). Applying non-invasive ventilation (NIV) with an expiratory positive airway pressure (EPAP) matching PEEPi improves gas exchange, reduces work of breathing and ineffective efforts. We aimed to evaluate the effects of a novel NIV mode that continuously adjusts EPAP to the minimum level that abolishes EFL_T_.

**Methods:**

This prospective, cross-over, open-label study randomized patients to one night of fixed-EPAP and one night of EFL_T_-abolishing-EPAP. The primary outcome was transcutaneous carbon dioxide pressure (*P*tcCO_2_). Secondary outcomes were: peripheral oxygen saturation (SpO_2_), frequency of ineffective efforts, breathing patterns and oscillatory mechanics.

**Results:**

We screened 36 patients and included 12 in the analysis (age 72 ± 8 years, FEV1 38 ± 14%Pred). The median EPAP did not differ between the EFL_T_-abolishing-EPAP and the fixed-EPAP night (median (IQR) = 7.0 (6.0, 8.8) cmH_2_O during night vs 7.5 (6.5, 10.5) cmH_2_O, p = 0.365). We found no differences in mean *P*tcCO_2_ (44.9 (41.6, 57.2) mmHg vs 54.5 (51.1, 59.0), p = 0.365), the percentage of night time with *P*tcCO_2_ > 45 mm Hg was lower (62(8,100)% vs 98(94,100)%, p = 0.031) and ineffective efforts were fewer (126(93,205) vs 261(205,351) events/hour, p = 0.003) during the EFL_T_-abolishing-EPAP than during the fixed-EPAP night. We found no differences in oxygen saturation and lung mechanics between nights.

**Conclusion:**

An adaptive ventilation mode targeted to abolish EFL_T_ has the potential to reduce hypercapnia and ineffective efforts in stable COPD patients receiving nocturnal NIV.

*Trial registration:* ClicalTrials.gov, NCT04497090. Registered 29 July 2020—Retrospectively registered, https://clinicaltrials.gov/ct2/show/NCT04497090.

## Background

We have recently introduced a novel automatic ventilation mode that continuously titrates expiratory positive airway pressure (EPAP) to the lowest value that abolishes tidal expiratory flow limitation (EFL_T_) [1]. This method, which uses the difference between inspiratory and expiratory reactance (ΔXrs) measured by the forced oscillation technique (FOT) [2, 3] to assess the presence of EFL_T_, minimizes the neural respiratory drive and trans-diaphragmatic pressure swings in COPD patients receiving non-invasive ventilation (NIV) [4].

We hypothesized that this adaptive ventilation mode would reduce hypercapnia during sleep in COPD patients with chronic hypercapnic respiratory failure. Moreover, since EFL_T_ is associated with the development of intrinsic positive end-expiratory pressure (PEEPi)—which acts as an inspiratory threshold for the generation of inspiratory flows and can produce ineffective breath triggering [5]—we further hypothesized that the EFL_T_-abolishing respiratory support mode would reduce the triggering load imposed by PEEPi reducing the probability of ineffective efforts.

This pilot study aimed to evaluate the effects of a novel ventilation mode that automatically adjusts EPAP at the minimum level able to abolish EFL_T_ compared to the standard fixed-EPAP mode in stable COPD patients receiving nocturnal NIV.

## Materials and methods

### Study design

In this prospective, randomized, cross-over, open-label pilot study, patients were studied in the hospital over two non-consecutive nights while using either fixed-EPAP or EFL_T_-abolishing-EPAP.

### Population

We enrolled moderate to severe COPD patients [6], with FEV1 ≤ 50%Pred, a history of more than 3 exacerbations per year or more than 1 hospitalization per year. Patients were well established on nocturnal NIV for chronic hypercapnic respiratory failure for longer than 6 months. Inclusion criteria were age below 85 years and presence of EFL_T_ in the supine position at EPAP = 4 cmH_2_O [1]. Exclusion criteria were COPD exacerbation within the past two months, acute illness, or clinical instability.

### Outcomes

The primary outcome was *P*tcCO_2_, expressed as mean overnight value and the percentage of night time spent in hypercapnia. Secondary outcomes were oxygen saturation, ineffective efforts, breathing pattern, and oscillatory mechanics. We hypothesized that mean *P*tcCO_2_ and the percentage of the night spent in hypercapnia would be lower during the EFL_T_-abolishing-EPAP than during the fixed-EPAP night.

### Ventilation strategy

Pressure support NIV was delivered using a non-commercial version of BiPAP Synchrony Ventilator (Philips-Respironics) via an unvented facial mask (AMARA, Philips-Respironics). The ventilator measured EFL_T_ by FOT [1, 2, 7] and, in EFL_T_-abolishing-EPAP mode, it continuously adjusted EPAP to the minimum level able to abolish EFL_T_ [1], with a minimum EPAP of 4 cmH_2_O and keeping the pressure support (∆P) constant.

### Measurements

Full laboratory polysomnography (Alice5, Philips-Respironics) was performed according to the American Academy of Sleep Medicine recommendations [8]. During each study night, we recorded *P*tcCO_2_ and oxygen saturation (SpO_2_) (TOSCA, Radiometer) continuously. Airway opening pressure, flow and volume tracings were exported from the ventilator for offline analysis. We calculated the following parameters: mean *P*tcCO_2_ and SpO_2_; the percentage of night time spent in hypercapnia (*P*tcCO_2_ > 45 mm Hg) and with SpO_2_ < 90% (T90); mean tidal volume (V_T_), respiratory rate (RR), minute ventilation (V_E_), inspiratory resistance and reactance (R_INSP_ and X_INSP_, respectively), ΔXrs, and the number of ineffective efforts (IE) per hour. We identified ineffective efforts by the presence of a positive deflection in expiratory flow without a concomitant breath delivered by the ventilator, as previously described [9]. At the end of the night, we asked the patients to report about their comfort on the ventilator.

### Statistical analysis

We compared parameters from the two nights using Wilcoxon signed-rank test. p-Values < 0.05 were considered statistically significant. Statistical analyses were performed using SigmaPlot v11 (Systat Software, Inc., San Jose, CA, USA).

## Results

**Fig. 1:**
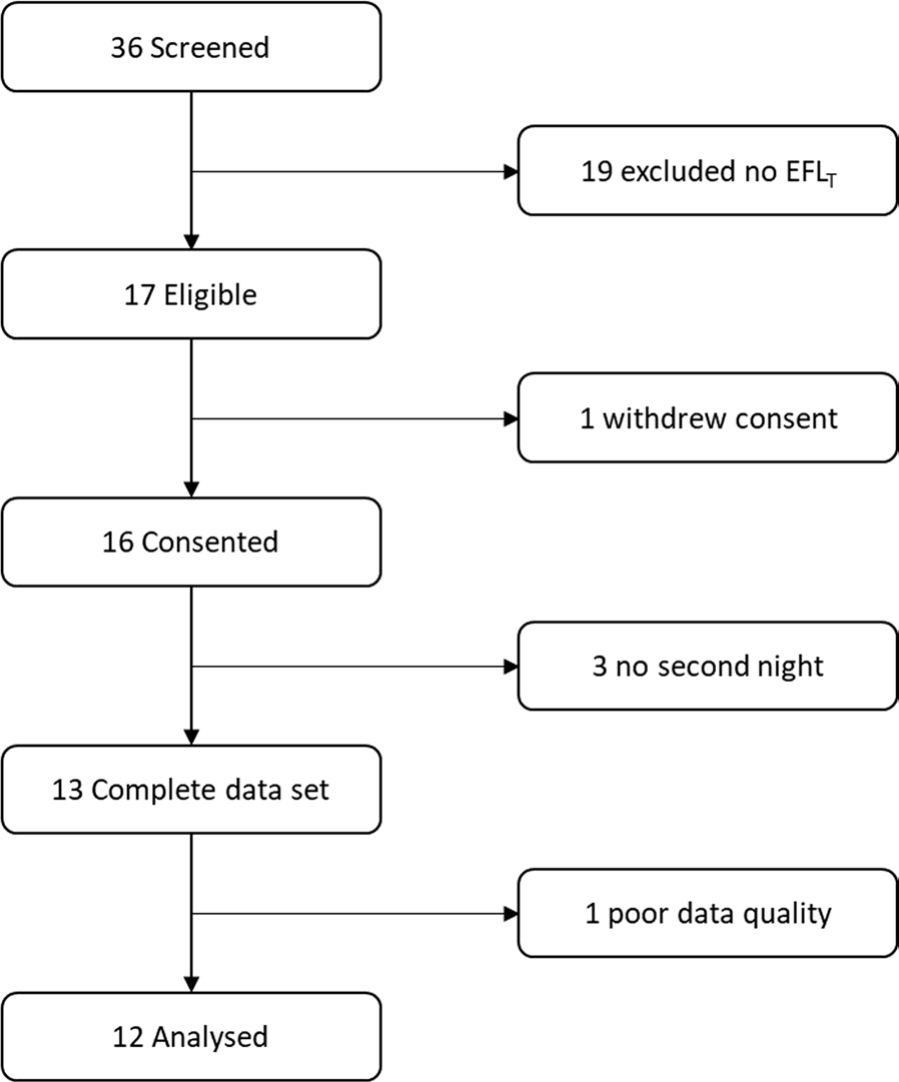
Study flow diagram. *EFL*_*T*_ tidal expiratory flow limitation

We screened thirty-six patients from April 2015 to April 2017. Of these patients, 19 did not satisfy the inclusion criteria as they did not present EFL_T_, one withdrew consent after the screening, two did not perform the second night trial because they got acutely sick, one was excluded from the analysis because of poor data quality, and 12 were included in the analysis (Fig. 1). Table 1 reports the characteristics of the patients included in the analysis.

**Table 1:** Characteristics of study participants

N	12
Sex, %males	67%
Age, years	73 (66, 79)
BMI, kg/m^2^	29.2 (27.7, 32.9)
FEV1, % predicted	33 (25, 47)
FEV1/FVC, %	43 (32, 60)
Prescribed EPAP, cmH_2_O	7.5 (6.0, 9.0)

*BMI* body mass index, *FEV1* forced expiratory volume in one second, *FVC* forced vital capacity, *EPAP* expiratory positive airway pressure

**Fig. 2:**
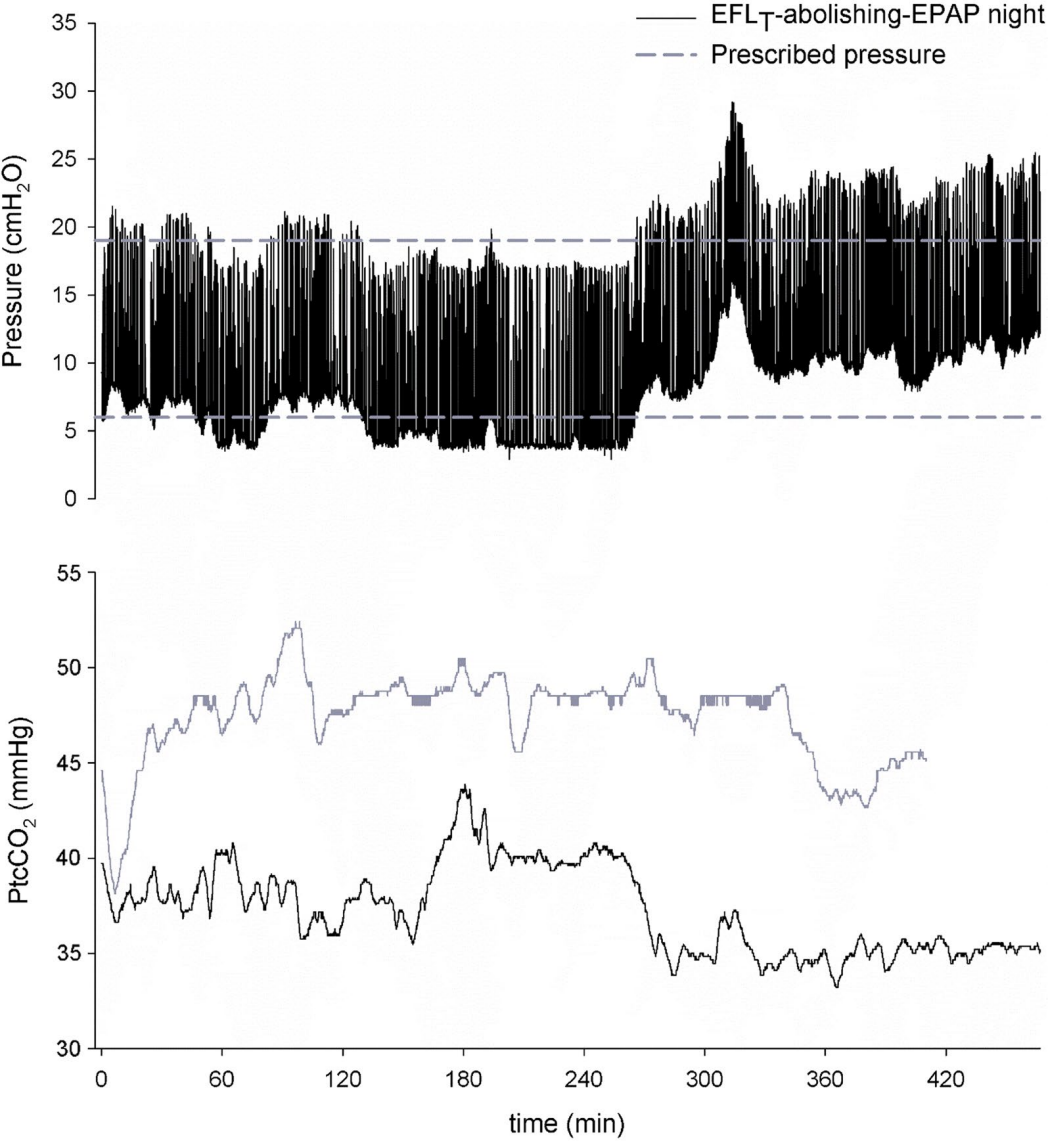
Representative pressure and *P*tcCO_2_ tracings for the EFL_T_-abolishing-EPAP (black) and the fixed-EPAP nights (grey). During the fixed-EPAP night, EPAP and IPAP were kept constant at the prescribed values (horizontal dashed lines)

Some patients acknowledged the presence of the oscillations, but they got acclimated after just few minutes. No patients reported discomfort during the EFL_T_-abolishing-EPAP night. We observed large within-night fluctuations in EPAP during the EFL_T_-abolishing-EPAP night: the minimum within-night IQR was 1.8 cmH_2_O, the maximum within-night IQR was 8.8 cmH_2_O. Figure 2 shows the airway pressure and *P*tcCO_2_ of a representative patient during both nights.

**Fig. 3:**
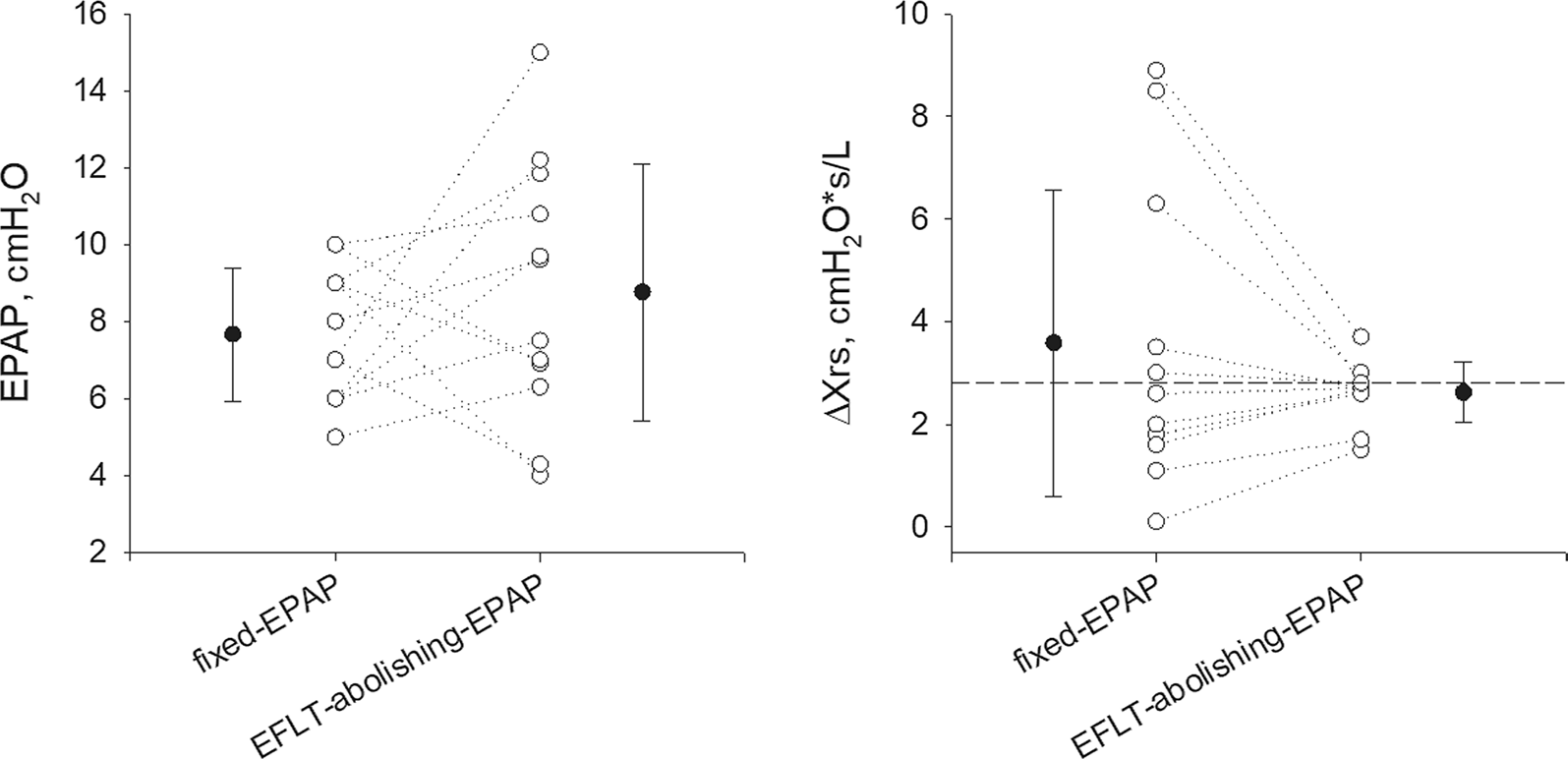
EPAP and tidal expiratory flow limitation index during the night with EFL_T_-abolishing-EPAP vs. fixed-EPAP. Data are reported as individual values (open symbols dotted lines) and as mean ± SD of all subjects (closed symbols). Dashed-line: tidal expiratory flow limitation threshold. EPAP: expiratory positive airway pressure. ΔXrs: difference between mean inspiratory and expiratory reactance (tidal expiratory flow limitation index). ΔXrs data for 11 subjects

The EPAP applied by the EFL_T_-abolishing mode was not significantly different from the prescribed EPAP (median (IQR) 7.0 (6.0, 8.8) cmH_2_O during the EFL_T_-abolishing-EPAP night vs 7.5 (6.5, 10.5) cmH_2_O during the fixed-EPAP night, p = 0.365), despite its larger within-night variability. ΔXrs values clustered around the EFL_T_ threshold during the EFL_T_-abolishing-EPAP night (Fig. 3).

**Fig. 4:**
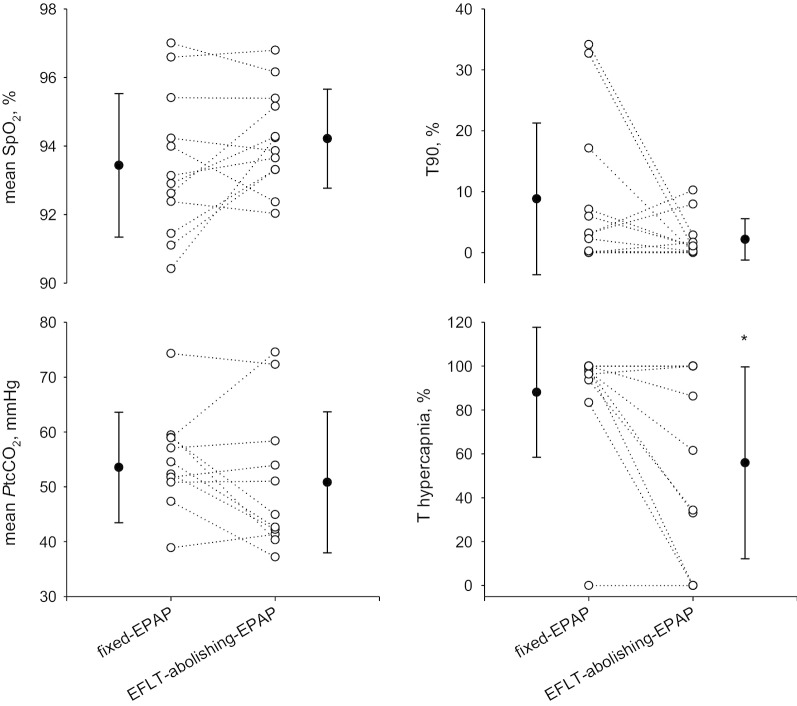
Gas exchange during the night with EFL_T_-abolishing-EPAP vs. fixed-EPAP. Data are reported as individual values (open symbols dotted lines) and as mean ± SD of all subjects (closed symbols). SpO_2_: peripheral oxygen saturation. T90: percentage of time spent with oxygen saturation SpO_2_ below 90%. *P*tcCO_2_: transcutaneous partial pressure of carbon dioxide. T hypercapnia: percentage of time spent with a *P*tcCO_2_ > 45 mm Hg. T hypercapnia and ΔXrs data for 11 subjects. *p < 0.05 between nights

Figure 4 shows gas exchange parameters during the EFL_T_-abolishing-EPAP and the fixed-EPAP nights. The percentage of time spent in hypercapnia was lower (median (IQR) = 62 (8, 100)% vs. 98 (94, 100)%, p = 0.031) during the EFL_T_-abolishing-EPAP than during the fixed-EPAP night. We found no differences in mean *P*tcCO_2_ between the EFL_T_-abolishing-EPAP and the fixed-EPAP night (44.9 (41.6, 57.2) mmHg vs 54.5 (51.1, 59.0), respectively; p = 0.365). Mean SpO_2_ and T90 did not differ between nights.

The IE were fewer (126 (93, 205) vs. 261 (205, 351) events/hour, p = 0.003) during the EFL_T_-abolishing-EPAP than during the fixed-EPAP night. Additionally, mean V_T_ was lower (p = 0.029), and RR was higher (p = 0.035) during the EFL_T_-abolishing-EPAP than during the fixed-EPAP night. V_E_, R_INSP_, and X_INSP_ did not differ between nights, even if the statistical power was too low to exclude an effect of the ventilation mode on these variables (Table 2).

**Table 2:** Respiratory parameters and sleep quality during the night with fixed vs. EFL_T_-abolishing-EPAP

	Fixed-EPAP	EFL_T_-abolishing-EPAP	p
*Gas exchange*			
mean *P*tcCO_2_, mmHg	54.5 (51.1, 59.0)	44.9 (41.6, 57.2)	0.365
**T hypercapnia, %**	**98 (94, 100)**	**62 (8, 100)**	**0.031**
Mean SpO_2_, %	93 (92, 95)	94 (93, 95)	0.204
T90, %	3 (0, 12)	1 (0, 2)	0.175
*Respiratory parameters*			
R_INSP_, cmH_2_O*s/L	9.7 (8.5, 12.0)	11.4 (9.3, 12.1)	0.577
X_INSP_, cmH_2_O*s/L	− 3.6 (− 4.1, − 2.3)	− 3.8 (− 3.9, − 2.9)	0.898
IPAP, cmH_2_O	15.7 (14.0, 17.1)	16.1 (14.3, 18.1)	0.638
**V**_**T**_**, mL**	**356 (252, 542)**	**272 (230, 395)**	**0.032**
**RR, bpm**	**16 (13, 18)**	**17 (14, 20)**	**0.019**
V_E_, L/min	5.3 (3.9, 6.4)	4.6 (3.8, 5.8)	0.123
**IE, n/h**	**261 (205, 351)**	**126 (93, 205)**	**0.003**

Data are reported as median (IQR)

Bold denotes a statistically significant difference between the fixed-EPAP and the EFL_T_-abolishing-EPAP nights (Wilcoxon signed-rank test, p < 0.05)

*PtcCO*_*2*_ transcutaneous partial pressure of CO_2_, *T hypercapnia* percentage of time spent with a transcutaneous partial pressure of CO_2_ > 45 mm Hg, *SpO*_*2*_ peripheral oxygen saturation, *T90* percentage of time spent with oxygen saturation SpO_2_ below 90%, *R*_*INSP*_ inspiratory resistance, *X*_*INSP*_ inspiratory reactance, *IPAP* inspiratory positive airway pressure, *V*_*T*_ tidal volume, *RR* respiratory rate, *V*_*E*_ minute ventilation, *IE* ineffective efforts

*p < 0.05 vs. fixed-EPAP

## Discussion

This is the first report of the nocturnal application of an adaptive NIV mode that continuously adjusts EPAP to the minimum level that abolishes EFL_T_ in hypercapnic COPD patients. This ventilation mode was well tolerated, reduced the frequency of ineffective efforts and the percentage of night time spent in hypercapnia. We found no differences in R_INSP_, X_INSP_, and ΔXrs between the two modes. The median EPAP did not significantly differ between nights; however, on an individual basis, some patients received significantly different (either higher or lower) EPAP levels during the EFL_T_-abolishing-EPAP and the fixed-EPAP nights. During the EFL_T_-abolishing-EPAP night, the EPAP presented large fluctuations, suggesting that the ventilator automatically adapted the EPAP level to the changes in lung mechanics associated with changes in posture and sleep stage.

The individual responses to abolishing EFL_T_ were highly heterogeneous, and this heterogeneity may have contributed to the lack of statistically significant differences in gas exchange between nights. In several subjects, we noticed a clinically relevant improvement in either *P*tcCO_2_ or SpO_2_ during the EFL_T_-abolishing-EPAP night. One patient presented a markedly higher mean *P*tcCO_2_ during the EFL_T_-abolishing-EPAP than during the fixed-EPAP night. This patient was very flow-limited, received a median EPAP of 12 cmH_2_O during the EFL_T_-abolishing-EPAP night vs 6 cmH_2_O during the fixed-EPAP night, and presented a much higher V_E_ during the EFL_T_-abolishing-EPAP than during the fixed-EPAP night. We did not identify any parameter able to predict the gas exchange response of a given patient to the EFL_T_-abolishing ventilation mode. Larger studies are needed to draw conclusions about the clinical benefits of this novel adaptive mode and to identify phenotypes that may better benefit from it.

NIV is used in stable COPD patients with hypercapnic respiratory failure to reduce arterial partial pressure of CO_2_ [10]. In our study improvements in *P*tcCO_2_ and in the percentage of night time spent in hypercapnia were not associated with increases in pressure support or V_E_, highlighting the relevant role of EPAP in the control of hypercapnia in COPD patients. Titrating EPAP to abolish EFL_T_ may reduce CO_2_ via several mechanisms [5, 11, 12]: (1) reducing work of breathing by improving patient-ventilator synchronization, (2) unloading the inspiratory muscles by counteracting the intrinsic PEEP, (3) reducing the ventilation-perfusion mismatch by eliminating choke-points. Moreover, EFL_T_ is highly variable within the same patient, e.g. it changes with body posture [1] and sleep stage. Therefore, an adaptive ventilation mode that continuously adjusts EPAP based on patient respiratory mechanics increases the time spent with the optimal EPAP compared with a fixed-EPAP mode, even if the average EPAP applied by the two ventilation modes is similar.

This study has several limitations. Since it was a short-term study, we could not assess long-term effectiveness and safety. Moreover, this was a pilot study on a small number of patients. Ten to fifteen patients is the typical sample size for pilot studies. This number is not calculated on statistical bases, but it is appropriate to assess the feasibility of a new method, inform possible improvements and collect preliminary data for larger clinical trials. The most reliable method for the assessment of ineffective efforts is to identify tidal swings in trans-diaphragmatic pressure (measured by gastric and oesophageal probes) that are not followed by an assisted breath. Our definition of ineffective efforts may have missed the efforts that did not generate any deflation in the flow signal, underestimating the actual number of events. However, we preferred not to measure the trans-diaphragmatic pressure because it could have disrupted sleep. We used *P*tcCO_2_ and pulse oximetry as indicators of gas exchange. Arterial blood gas measurements would have been more precise; however, in the ordinary setting of polysomnography, it is not possible to have an invasive continuous measurement of arterial blood gasses. On the other hand, single blood gas measurements are not representative of the gas exchange during sleep, and multiple overnight assessments usually determine sleep disruption.

## Conclusion

In conclusion, the use of a NIV mode that continuously auto-titrates EPAP to abolish EFL_T_ during sleep has the potential to control hypercapnia better and reduce ineffective efforts compared with fixed-EPAP modes in COPD. Larger studies are needed to draw conclusions about the clinical benefits of this novel ventilation mode and to assess its long term effects.

## Data Availability

The datasets used and analyzed during the current study are available from the corresponding author on reasonable request.

## Abbreviations

COPD: Chronic obstructive pulmonary disease
EPAP: Expiratory positive airway pressure
EFL_T_: Tidal expiratory flow limitation
FOT: Forced oscillation technique
IE: Number of ineffective efforts per hour
ΔXrs: Difference between mean inspiratory and expiratory reactance
NIV: Non-invasive ventilation
*P*tcCO_2_: Transcutaneous partial pressure of carbon dioxide
R_INSP_: Mean inspiratory resistance
RR: Respiratory rate
SpO_2_: Peripheral oxygen saturation
T90: Percentage of night time with SpO_2_ < 90%
V_E_: Minute ventilation
V_T_: Tidal volume
X_INSP_: Inspiratory reactance

## References

[1] Milesi I, Porta R, Barbano L, Cacciatore S, Vitacca M, Dellacà RL (2019). Automatic tailoring of the lowest PEEP to abolish tidal expiratory flow limitation in seated and supine COPD patients. Respir Med.

[2] Dellacà RL, Santus P, Aliverti A, Stevenson N, Centanni S, Macklem PT (2004). Detection of expiratory flow limitation in COPD using the forced oscillation technique. Eur Respir J.

[3] Zannin E, Chakrabarti B, Govoni L, Pompilio PP, Romano R, Calverley PMA (2019). Detection of expiratory flow limitation by forced oscillations during noninvasive ventilation. Am J Respir Crit Care Med.

[4] Suh E-S, Pompilio P, Mandal S, Hill P, Kaltsakas G, Murphy PB (2020). Auto-titrating external positive end-expiratory airway pressure to abolish expiratory flow limitation during tidal breathing in patients with severe chronic obstructive pulmonary disease: a physiological study. Eur Respir J.

[5] Fanfulla F, Taurino AE, Lupo NDA, Trentin R, D’Ambrosio C, Nava S (2007). Effect of sleep on patient/ventilator asynchrony in patients undergoing chronic non-invasive mechanical ventilation. Respir Med Respir Med.

[6] Vogelmeier CF, Criner GJ, Martinez FJ, Anzueto A, Barnes PJ, Bourbeau J (2017). Global strategy for the diagnosis, management, and prevention of chronic obstructive lung disease 2017 report. Am J Respir Crit Care Med.

[7] Dellacà RL, Rotger M, Aliverti A, Navajas D, Pedotti A, Farré R (2006). Noninvasive detection of expiratory flow limitation in COPD patients during nasal CPAP. Eur Respir J.

[8] American Academy of Sleep Medicine (2007). The AASM manual for the scoring of sleep and associated events: Rules, terminology and technical specifications.

[9] Fanfulla F, Delmastro M, Berardinelli A, Lupo NDA, Nava S (2005). Effects of different ventilator settings on sleep and inspiratory effort in patients with neuromuscular disease. Am J Respir Crit Care Med.

[10] Ergan B, Oczkowski S, Rochwerg B, Carlucci A, Chatwin M, Clini E (2019). European Respiratory Society guidelines on long-term home non-invasive ventilation for management of COPD. Eur Respir J.

[11] Appendini L, Purro A, Patessio A, Zanaboni S, Carone M, Spada E (1996). Partitioning of inspiratory muscle workload and pressure assistance in ventilator-dependent COPD patients. Am J Respir Crit Care Med.

[12] Elliott MW, Mulvey DA, Moxham J, Green M, Branthwaite MA (2007). Inspiratory muscle effort during nasal intermittent positive pressure ventilation in patients with chronic obstructive airways disease. Anaesthesia.

